# Revolutionizing smart grid security: a holistic cyber defence strategy

**DOI:** 10.3389/frai.2024.1476422

**Published:** 2024-12-03

**Authors:** Bhushankumar Nemade, Kiran Kishor Maharana, Vikram Kulkarni, Ch Srivardhankumar, Mahendra Shelar

**Affiliations:** ^1^Department of CSE, Shree L.R. Tiwari Engineering College, Mumbai University, Mumbai, Maharashtra, India; ^2^ICICI Lombard GIC Ltd, Mumbai, Maharashtra, India; ^3^Department of Information Technology, Mukesh Patel School of Technology, Management, and Engineering, SVKM's NMIMS, Mumbai, Maharashtra, India; ^4^Department of EEE, MLR Institute of Technology, Hyderabad, Telangana, India; ^5^Department of Mechanical Engineering, Thakur College of Engineering and Technology, Mumbai, Maharashtra, India

**Keywords:** smart grid, cyber-security, vulnerability, deep learning, anomaly detection, threats, vulnerabilities, SCADA systems

## 1 Introduction

The country's energy infrastructure is a national asset inextricably linked to national progress (Kulkarni et al., 2024). Old grids are stiff, fail to balance loads, and have a significant risk of cascading failures, making them unsuitable for current times (Ravinder and Kulkarni, 2023b). Other difficulties include interoperability and scalability, high costs, data privacy, and security. They also face legacy system dependencies, regulatory and compliance issues due to outmoded capabilities (Bouramdane, 2023). Transitioning to a smart grid enables dynamic solutions for load management, self-healing capabilities, and decentralized decision-making.

As smart grids help us move away from legacy issues, the inclusion of new-generation technology makes the system prone to cyberattacks. In many developed and developing countries, smart grids bring hope for strengthening the sector by providing clean energy that meets future goals of both the political and economic classes. However, when negligence occurs while introducing these futuristic systems, they usually result in inheriting legacy issues along with vulnerabilities arising from cyberspace. In the context of Indian institutions, they often demonstrate a weak approach and inefficient environments, making them susceptible to attacks by adversaries on their energy sector (Ravinder and Kulkarni, 2023c).

### 1.1 Intrusion into Nation's critical infrastructure

Espionage, an ancient form of warfare, becomes particularly lethal for an individual citizen and country as a whole, when combined with individually motivated attackers. Between 2021 and 2022, there were reports of Chinese government-linked hackers attempting to infiltrate and steal data from the Indian government as well as major players within the Indian power sector (Bouramdane, 2023). In 2019, Venezuela struggled with the attack not only on its technical aspects but also through cyberattacks, leaving the country in prolonged blackouts (Ravinder and Kulkarni, 2023c). Similarly, in 2015, Russian hackers targeted Ukraine's power grid^1^, a pattern that continued during the 2023–2024 conflict between the two nations.

These attacks are often well-coordinated, synchronized, and executed with a high level of professionalism, leading to day-long outages. The effects go beyond economic losses, which range from millions to billions of dollars, and imperil lives.

### 1.2 Threats and vulnerabilities

All of these precautions necessitate an understanding of the most prevalent security vulnerabilities that smart grids face, which can have serious consequences for their operation and integrity:

- Network attacks: these mostly target network operators, power plants, and utility businesses. This is to disrupt utility delivery while causing disruption and potentially obtaining ransom payments.
- Breaching sensitive customer data: sensitive client data may be compromised by adversaries, so posing significant privacy concerns.
- Malware propagation: malware can readily permeate smart grid systems, potentially affecting operations and causing widespread disruption.
- Distributed control devices: according to reports, attackers exploit vulnerabilities in distributed control devices to take over or impair grid operations without authorization.

Smart grids have varying degrees of vulnerability; however, they are susceptible to many types of attacks:

- At the consumer access level, smart meters have the disadvantage of serving as a gateway for collecting and transmitting data about energy consumption. These meters, if infiltrated, would represent serious breaches of privacy or illegal access, allowing attackers to tamper with data or even disrupt services.
- Another highly sought-after target site is the communication network level, which supports grid communication, whether through wireless networks (Wang and Lu, 2013). Here, attacks might disrupt data transit and jeopardize grid control, resulting in operational pandemonium. Such attacks on SCADA systems, which are crucial to grid control, have the ability to destabilize and manipulate grid functionalities.
- Decisions are made at the utility company and operator levels. A utility company's system may fail, causing widespread disruptions in electricity distribution for customers and businesses. This type of multi-layered vulnerability framework has been shown to necessitate comprehensive security designs to protect smart grids from numerous and changing threats.

Furthermore, attacks against smart grids can be classified based on OSI model levels, presenting the various types of potential vulnerabilities (Mughal, 2020; Patel et al., 2016):

- Application layer: malware designed for scada software or smart grid applications that aims to interrupt or steal data.
- Presentation layer: steal encryption protocol keys to intercept or modify sensitive data.
- Session layer: sessions can be hijacked to gain unauthorized control over communication sessions in the grid.
- Transport layer: hacking TCP connections can impede data flow and obtain access to grid control systems.
- Network layer: DoS attacks on routers or switches can disrupt data flows and communication.
- Data link layer: using MAC spoofing to enter and change data flow without permission.
- Physical layer: physically interfering with substations or power cables to cause outages or damage.

It indicates that a deeper knowledge of these threats and vulnerabilities is critical to improving the security and resilience of smart grids and ensuring dependable operation while preventing potential disruptions.

## 2 Detection technique—Holistic cyber defence interaction

With the introduction of new methods and appropriate procedures, the system becomes more complex in its ability to safeguard itself. However, it is critical to understand that proper approach implementation is always necessary.

A PSU or a large organization requires many recurring and multiple permissions, such as raising tickets for accessing ports with common channels, which can be critical to assuring security and compliance across organizations. But these processes often have numerous steps, such as risk assessments, security evaluations, and managerial approvals. However, the complexity and length of these processes usually results in delay resulting in irritation and frustration towards it among developers, architects, and other critical team players working on the project. This implies that such actors will need and ask for faster solutions that are or doesn't include bureaucratic red tape that might inadvertently introduce security vulnerabilities.

The Holistic Cyber Defence Interaction (HCDI) technique introduction can solve these difficulties by creating a collaborative environment in which the entire business works together to develop the best answers. HCDI aims to combine human-AI interaction with powerful Deep Learning (DL) and graph-based algorithms to ensure that security measures are resilient, comprehensive, efficient, and streamlined. This would, to an extent, bring uniformity in the process resulting in decrease the number of approvals as the processes of risk assessment and security review are automated. These claims are on the basis of the Policy and Mechanisms written. Thus, making it less prone to human error and omission. HCDI will enable organizations to sustain robust cyber strengths while still maintaining pace and efficiencies in operations through a multi-dimensional concerted effort.

The HCDI represents a pioneering approach that synergistically combines advanced methodologies from semi-supervised anomaly detection, deep representation learning, graph-based specification analysis, adaptive real-time detection, and deep learning ensembles with attention mechanisms. It is designed in such a way that it bring enhancement of cybersecurity in smart grids and simplification in terms of robust Policies and Policy mechanisms to implement them without any leaks.

### 2.1 Implementation and impact

The HCDI is a like wrapper around the best available Frameworks today. It focuses from the base to upliftment of the pillar. It includes and takes motivation from several available Frameworks which starts from data collection to development to scalability.

Implementing HCDI involves several key steps to ensure effective deployment and integration within smart grid cybersecurity frameworks.

### 2.2 Data collection and preparation

To train anomaly detection models effectively for smart grids, datasets to be gathered must represent diverse operational data collected from various components of the smart grid, such as substations and SCADA systems. Some of the points to be considered include:

1. Diverse operational scenarios: datasets should represent various operational scenarios, including peak and off-peak hours, maintenance periods, different weather conditions, few to be mentioned. It is important to have such diversity in the features available in the dataset as it will help to create a robust model that can handle many real-world variabilities.
2. Historical data: having historical data in the collection is necessary to understand and capture long-term trends and patterns. Exploratory Data Analysis (or commonly abbreviated as EDA) for understanding the operational behaviour and identify the outliers or any important deviation which can help understand anomalies and limitation in the legacy systems.
3. Formulating policies and mechanisms: before moving ahead from these steps it is important to formulate policies or what and what is not allowed. The mechanisms should be built around it such that it implements the policies without any gaps left out. The crucial step should also include a phase to maintain the access control matrix.
4. Real-time data integration: build mechanisms for real-time data gathering and integration. This will help in continuous updating of the dataset. This will be necessary and helpful for bringing in new operational data and newly detected anomalies, thus enhancing the adaptability of the model to the changing threats.
5. Anomaly authorization: use a mechanism for approving and verifying recently discovered anomalies before adding them to the dataset. This will guarantee that model training uses only pertinent and validated abnormalities, enhancing the accuracy and dependability of the detection system.
6. Data sources: making use of data from many sources inside the smart grid—such as sensors, meters, and communication networks—allows one to create This multi-source method will give a full picture of the operational situation on the grid. We should also consider additional factors that could lead to data source corruption and consequent loss of relevance for model development.
7. Data quality and preprocessing: ensuring high data quality by addressing issues such as missing values, noise, and inconsistencies. Preprocessing steps like normalization and feature extraction are to be taken in consideration before training the model. Further removing and reducing size of the dataset features with various ML techniques for better performance and low cost. This can be done by Feature Extraction and Feature Dimensionality Reduction.

By following these guidelines and modification as per the use case, it helps to create a robust and comprehensive dataset that effectively supports the development of accurate and reliable smart grid anomaly detection model

### 2.3 Model development

Semi-supervised anomaly detection with deep representation learning: development of autoencoder models for creating different layers of learning and representation of normal data patterns. Training models based on normal operational data excluding anomalies. While testing validate are the models/model able to detected the anomalies able to point to potential cyber-attacks (Qi et al., 2021).

Graph-based specification: development of a graph representation of the smart grid infrastructure, with nodes representing critical components and edges denoting relationships. Defining expected graph structures and applying graph analysis techniques to detect deviations and outliers that might results or indicate cyber intrusions (Klaer et al., 2020).

Adaptive cumulative sum (CUSUM) for real-time detection: integrate CUSUM algorithms into SCADA systems or data processing pipelines. Configure CUSUM to monitor real-time data streams, adapt to changing load behaviours, and trigger alerts upon detecting anomalies (Olufowobi et al., 2019).

Deep learning ensembles with attention mechanisms: develop an ensemble of LSTM, CNN, and RNN models equipped with attention mechanisms. Train the ensemble on historical data to capture complex patterns and important features present indicative of cyber threats (Boopathy et al., 2024).

## 3 Integration and deployment

Integrating the developed models and algorithms into the given smart grid infrastructure and cybersecurity frameworks so that information communication is uniform and harmonious between the detection systems, SCADA systems, and the different responding mechanisms of the cybersecurity framework. Advanced AI and ML techniques such as real-time threat identification and prediction include anomaly detection, clustering, and deep learning.

TensorFlow and PyTorch can be used to create strong models based on Scikit-learn. Implement distributed computing platforms such as Apache Kafka and Apache Spark to process and analyze data efficiently. Additionally, use SIEM systems such as Splunk and ELK Stack for centralized collection of security data for further analysis and optimized response. From these tools and techniques, include support to further the overall resilience and responsiveness of the smart grid against cyber threats.

Table 1 summarizes the tools and CI/CD pipeline frameworks utilized for various components of the Smart Grid Architecture, highlighting their roles in enabling seamless integration, automation, and efficient management of smart grid functionalities.

**Table 1:** Tools and CI/CD pipeline for different components of smart grid architecture.

**Category**	**Tool**	**CI/CD pipeline tools**
AI/ML development	TensorFlow, PyTorch, Scikit-learn	Jenkins, GitLab CI/CD, CircleCI
Distributed computing	apache Kafka, apache spark	Kubernetes, Docker, Jenkins
SIEM Systems	Splunk, ELK stack	Jenkins, GitLab CI/CD, CircleCI
SCADA systems	Custom SCADA software	Jenkins, GitLab CI/CD, CircleCI
Cybersecurity frameworks	Custom security tools	Jenkins, GitLab CI/CD, CircleCI

Table 2 outlines the key code aspects, associated checkpoints, and the recommended tools to ensure adherence to coding standards, quality assurance, and efficient development practices within the Smart Grid Architecture framework.

**Table 2:** Code aspects with checkpoints and tools to use.

**Aspect**	**Checkpoints**	**Tools**
Code style and consistency	Ensure consistent code formatting and style	ESLint, Prettier, Pylint
Code complexity	Measure and manage code complexity	SonarQube, CodeClimate, JArchitect
Code coverage	Ensure code is well-tested	Jest, Mocha, JUnit, Istanbul
Code security	Identify and fix security vulnerabilities	SonarQube, Checkmarx, Snyk
Code performance	Analyze and improve code performance	JProfiler, VisualVM, New Relic
Code documentation	Document code for better understanding	JSDoc, Sphinx, Doxygen
Code review	Facilitate peer code reviews	GitHub Pull Requests, GitLab Merge Requests, Crucible
Code testing	Automated testing frameworks	Selenium, Cypress, JUnit, PyTest

### 3.1 Continuous monitoring and evaluation

Implement mechanisms for continuous monitoring of model performance and cybersecurity effectiveness. To adapt to the new cyber threats and changes in the operation of smart grids, follow routine assessments and updates. AI and ML may play a critical role if they could perform real-time analysis of enormous volumes of data, pattern recognition, and predict the possible threats (Bold Group, 2024).

Some techniques include the usage of clustering, anomaly detection, and supervised learning with Random Forests (RF), Support Vector Machine (SVM), and Neural Networks (NNs) (Nemade and Shah, 2023; Mishra et al., 2023). K-Means Clustering is unsupervised and can be used to trace unknown threats. Algorithmic approaches may also evolve over time, therefore adapting to new threat vectors as well as changes in operations for stronger and more up-to-date cybersecurity measures (Nemade and Shah, 2022a,b).

## 4 Team collaboration

Foster collaboration among cross-functional teams to enhance threat detection capabilities and response strategies (Ravinder and Kulkarni, 2023a).

Table 3 presents actionable insights for team collaboration, detailing strategies, tools, and best practices to enhance communication, coordination, and productivity within the Smart Grid Architecture development process.

**Table 3:** Team collaboration actionable.

**Action**	**Benefit**
Regular cross-functional meetings	Align objectives, share insights, and update on progress
Unified threat detection platforms	Centralize monitoring and data sharing
Incident response drills	Enhance coordination and readiness for real threats
Integrated communication tools	Facilitate real-time information exchange
Shared documentation and knowledge base	Ensure consistency and accessibility of information
Collaborative analytics and reporting	Enable comprehensive threat analysis and reporting
Joint security workshops and training	Build skills and awareness across teams

### 4.1 Compliance and governance

Therefore, companies should set up governance frameworks for access controls, data privacy, and response procedures to ensure that the requirements of regulatory compliance and industry standards about cybersecurity in smart grid operations are met (Powerline, 2024; Kaul et al., 2016). In light of their needs, companies should gain certifications on industrial and market pressures to supply the desired goods and services apart from the scope of their activities. The other burden of too many, unnecessary certifications on an organization is that they consume precious man-hours in the form of compliance documentation and understanding new requirements, rather than practical implementation of necessary security measures.

The certifications needed in India are ISO/IEC 27001, CERT-IN Guidelines, IEC 62443, the Cybersecurity Framework provided by the Bureau of Indian Standards (BIS), and compliance with the Indian Electricity Grid Code (IEGC) for secure operations of the smart grid. Additionally, international standards compliance can also be met through centralization and harmonization of teams that understand the information of compliance frameworks such as NERC CIP, SOC 2, C2M2, FISMA, and GDPR.

All personnel must also receive training in technologies related to their job as well as accreditations like Certified in Risk and Information Systems Control (CRISC), Certified Information Privacy Professional (CIPP), Certified Information Security Manager (CISM), Certified Information Systems Auditor (CISA), Certified Information Systems Security Professional (CISSP), GIAC Security Essentials (GSEC), CompTIA Security+, and cloud-specific accreditations, including Google Cloud—Professional Cloud Architect or AWS Certified Solution Architect (Nemade et al., 2011).

A compliant organization with skillful employees will make successful and sustained implementation and maintenance of cybersecurity measures for smart grids, ensuring a secured and resilient operation.

### 4.2 Scalability and adaptability

Design the implementation to be scalable across large-scale smart grid deployments. Plan for adaptation to evolving cyber threats and technological advancements in AI and cybersecurity.

Here's an example which outlines how AWS service and open-source tools can help here.

Table 4 provides an example illustrating the integration of AWS services with open-source tools, showcasing their complementary use in optimizing Smart Grid Architecture deployment and management.

**Table 4:** Example outlining AWS and open-source tools integration.

**Service/tool**	**Function**	**Benefit**
Amazon EC2	Compute resources, application deployment	Scalable compute capacity, dynamic resource allocation
Amazon S3	Data storage	High durability, availability, and scalability of data
Amazon RDS	Relational database management	Automated backups, patching, and scaling
Amazon CloudFront	Content delivery network (CDN)	Reduced latency, efficient handling of traffic spikes
AWS lambda	Event-driven processing	Automatic scaling, cost efficiency (pay-per-use)
Amazon kinesis	Real-time data streaming and analytics	Real-time insights, handling large volumes of data
Amazon VPC	Network isolation and security	Network segmentation, secure communication
AWS IoT core	IoT device management and secure communication	Secure, scalable IoT device management
GridAttackSim	Cyber-attack simulation on smart grid infrastructure	Visualization of attack consequences, security assessments
OMNET++ and ns-3	Communication network simulation	Evaluation of communication protocols
OpenDSS	Power system simulation	Analysis of power distribution system resilience
IEEE C37.118	Synchrophasor data communication standard	Secure communication between smart grid devices

Such procedures of implementation undertaken by the companies will ensure the successful integration of HCDI into businesses, smart grid security posture would increase, reduce the complexity to the approval process, and let effective collaboration prevail over cyber threats.

This encapsulates the approach, that is hybrid or combined, which calls for advanced and legacy frameworks to unite and be driven by AI and ML to result in a resilient and elastic cybersecurity solution for smart grids. This strategy assures resilience against changing cyber threats and continuous improvement.

## 5 Conclusion

This actually is an important step towards energy management, but simultaneously threatens critical infrastructure to potential advanced cyberattacks. In this regard, it is emphasized by the current study that the practice of extensive cybersecurity measures, in particular HCDI technique, should be obligatory. Thus, by integrating real-time detection with graph-based analysis, deep learning, the HCDI framework stands as a strong base for smart grid security. HCDI ensures timely, effective incident response by expediting approval procedures and encouraging cross-functional cooperation. When HCDI is well implemented, smart grids become more resilient and reliable in protecting national energy infrastructures against threats.

## Footnotes

^1^ https://www.businesstoday.in/technology/news/story/did-chinese-hackers-target-indian-govt-air-india-reliance-everything-about-isoon-leak-418542-2024-02-22
